# Rush Medical College

**Published:** 1846-06

**Authors:** 


ARTICLE XV.

RUSH MEDICAL COLLEGE.

The annual announcement of this Institution is now in press, and will with the catalogue be distributed to the profession immediately. Since its first organization, this College has progressed in the confidence of the medical public, if the increase in the number of students can be taken as an index, more rapidly, it is believed, than any other western medical school at its commencement. The first class, three years since, numbered about twenty students, that in attendance during the past winter, numbered upward of fifty, showing an increase of one hundred and fifty per cent. It is a gratifying reflection that this increase has been brought about only by the advantages for improvement which the school offers to medical students.

It affords already the conveniences of a fine building; the supply of the material for dissecting is abundant; it has a good chemical apparatus, a cabinet of materia medica, and a mineralogical cabinet, while the means for illustrating healthy and morbid anatomy, by preparations, morbid specimens, and engravings, are ample. Surgery also in its principles and by operations upon the living and dead subjects, is fully taught. But the motto of the school is "progress," and efforts are now being made for adding greatly to the means. These additions will consist of a library embracing about 600 volumes of well selected and valuable medical works, calculated for reference and accessible to the students, but not intended for furnishing them with text books; of a set of drawings of large size, illustrating all the principal deformities, and dislocations. A hospital, where practical knowledge on all the various forms of disease may be witnessed, will also be established, affording to students an opportunity of becoming *practitioners.* With these advantages, and the care which will be taken to instil into the minds of students, elevated and honorable views of the profession and its duties, the Medical College of Chicago, it is believed, merits the confidence and support of the medical men in this region.

